# Antipsychotic polypharmacy and metabolic syndrome in schizophrenia: a review of systematic reviews

**DOI:** 10.1186/s12888-018-1848-y

**Published:** 2018-09-03

**Authors:** Sharea Ijaz, Blanca Bolea, Simon Davies, Jelena Savović, Alison Richards, Sarah Sullivan, Paul Moran

**Affiliations:** 1 Bristol Medical School, University of Bristol, Bristol, UK; 2 National Institute for Health Research (NIHR) Collaboration for Leadership in Applied Health Research and Care (CLAHRC) West, 9th floor, Whitefriars, Lewins Mead, Bristol, BS1 2NT UK; 3 Centre for Addiction and Mental Health (CAMH), University of Toronto, Toronto, Canada

**Keywords:** Schizophrenia, Antipsychotics, Diabetes mellitus, Metabolic syndrome

## Abstract

**Background:**

There is conflicting evidence on the association between antipsychotic polypharmacy and metabolic syndrome in schizophrenia. We conducted a review of published systematic reviews to evaluate evidence on the association between metabolic syndrome (diabetes, hypertension, and hyperlipidaemia) and exposure to antipsychotic polypharmacy in schizophrenia.

**Methods:**

We searched five electronic databases, complemented by reference screening, to find systematic reviews that investigated the association of antipsychotic polypharmacy in schizophrenia with hypertension, diabetes, or hyperlipidaemia. Selection of reviews, data extraction and review quality were conducted independently by two people and disagreements resolved by discussion. Results were synthesised narratively.

**Results:**

We included 12 systematic reviews, which reported heterogeneous results, mostly with narrative syntheses and without pooled data. The evidence was rated as low quality. There was some indication of a possible protective effect of drug combinations including aripiprazole for diabetes and hyperlipidaemias, compared to other combinations and/or monotherapy. Only one review reported the association between APP and hypertension. The most frequently reported combinations of medication included clozapine, possibly representing a sample of patients with treatment resistant illness. No included review reported results separately by setting (primary or secondary care).

**Conclusions:**

Further robust studies are needed to elucidate the possible protective effect of aripiprazole. Long-term prospective studies are required for accurate appraisal of diabetes risk, hypertension and hyperlipidaemia in patients exposed to antipsychotic polypharmacy.

**Electronic supplementary material:**

The online version of this article (10.1186/s12888-018-1848-y) contains supplementary material, which is available to authorized users.

## Background

Schizophrenia is a severe mental illness with a prevalence of approximately 1% [1].

It is expensive to treat [2] and at least 30% of patients with this illness experience a poor long-term prognosis, characterised by residual psychotic symptoms [3], poor social functioning and a poor quality of life [4]. People with schizophrenia die on average 20 years earlier than individuals without this illness and this gap is widening [5]. One of the possible explanations for the differential mortality rate is that patients with schizophrenia have an increased risk of metabolic syndrome such as diabetes, obesity, hypertension and hypercholesterolemia [6].

Antipsychotic medication is the first line treatment for schizophrenia [7, 8]. Antipsychotic drugs are effective for the treatment of the core symptoms of schizophrenia, such as auditory hallucinations and delusions. These drugs can be divided in two main classes: first generation antipsychotics (FGA or typical antipsychotics) such as haloperidol and second-generation antipsychotics (SGA or atypical) such as risperidone, olanzapine and quetiapine. FGAs are dopamine antagonists acting on the three main pathways for this neurotransmitter, while SGAs have in general, an affinity for both dopamine and 5HT _2_ receptors and are thought to be more selective towards the mesolimbic system [9]. Aripiprazole differs from other established atypical antipsychotics in being a partial agonist of dopamine receptors, and is considered by some authors to be sufficiently distinct to merit classification as a ‘third generation’ antipsychotic [10]. Schizophrenia is a chronic illness and most patients require lifelong treatment. Side-effects of antipsychotics accumulate over time. Long-term treatment with antipsychotic medication can increase the risk of diabetes, hypertension and hyperlipidaemia [11]. This state of metabolic change leading to an increased risk of cardiovascular and metabolic illness is known as metabolic syndrome. There are several definitions of metabolic syndrome [12]. The American Heart Association identifies six main components: abdominal obesity, dyslipidaemia, increased blood pressure, glucose intolerance and a pro-inflammatory and pro-thrombotic state [12]. In this overview, we use the term ‘metabolic syndrome’ to refer to the occurrence of hyperlipidaemia, diabetes or hypertension which are disorders commonly requiring treatment with medication.

Antipsychotic polypharmacy (APP) is defined as the simultaneous prescription of more than one antipsychotic medication. Patients may be prescribed more than one antipsychotic when they are deemed resistant to the effect of a single antipsychotic, they have more than one psychiatric diagnosis, the clinician is overlapping one medication while another is titrated, or an effective dose of one antipsychotic cannot be achieved because of lack of tolerance or side effects [8]. APP is not actively recommended in current clinical practice guidelines, yet it is extremely common in clinical practice, occurring in up to two-thirds of patients with psychosis [13–16].

APP is controversial because of a lack of clear evidence for treatment efficacy and the possible increased risk of side-effects over and above side-effects associated with anti-psychotic monotherapy [8, 17]. Research conducted mostly in secondary-care has produced conflicting evidence on the association between APP and metabolic syndrome, with some studies suggesting an increase and some a reduction in risk [13, 18, 19].

A scoping review conducted by this group suggested a need to collate the evidence from systematic reviews on the link between APP and metabolic syndrome to facilitate clinical decisions and stimulate new research in this area.

### Aims of the study

To conduct a review of published systematic reviews to assess the current state of the evidence on the association between antipsychotic polypharmacy (APP) used for the management of schizophrenia and metabolic syndrome (defined as diabetes, hypertension, or hyperlipidaemia).

## Methods

This review followed guidance published by the Centre for Reviews and Dissemination [20]. We wrote a protocol for the review with pre-specified objectives, eligibility criteria and review methods and registered it with PROSPERO (CRD42017054672) [21].

### Inclusion criteria for reviews

We included systematic reviews that reported an investigation of the association between APP and metabolic syndrome (diabetes, hypertension or hyperlipidaemia) in adults with schizophrenia treated in any setting. To be inclusive, we considered any reviews and meta-analysis reports to be systematic reviews as long as they reported a systematic search when evaluating the association between APP and metabolic syndrome.

We excluded reviews that focussed on animal or laboratory studies only.

### Identification and selection of reviews

Five databases (Medline, Embase, Cochrane, PsychInfo and Web of Science) were searched from inception until February 2017 to identify relevant reviews, using a systematic review filter. The search strategy for Medline is reported in the web appendix (Additional file 1). Searches were not limited by language, date, setting or publication status. An internet search using Google Scholar and screening reference lists of included publications were used to identify any additional relevant unpublished reviews. A systematic review filter was applied along with removal of duplicates to find relevant reviews. We did not search any regional databases.

Titles and abstracts of all citations from the search were independently screened by two reviewers and discrepant decisions resolved by discussion. Full text screening was then undertaken by two reviewers and disagreements resolved by a third reviewer with experience in psychopharmacology.

### Data extraction and quality assessment of included reviews

Data extraction and quality assessment were conducted in duplicate and disagreements resolved by discussion. We used a standardized data extraction template and extracted the following items from included reviews: country of study; funding source; number of studies included in review; dates of search; setting (primary/secondary care); designs of reviewed studies; whether a meta-analysis was conducted; types of participants, intervention, comparator, outcome and definition of outcome; whether a formal quality/risk of bias assessment was conducted and its findings; and results or findings of the review.

We used the validated AMSTAR (A MeaSurement Tool to Assess systematic Reviews) checklist [22] for assessing reporting quality of the systematic reviews included.

### Data synthesis

We carried out a narrative synthesis of the included systematic reviews with findings summarised in the text by outcome [20].

### Analysis of subgroups or subsets

We planned to investigate the effects of the combination of different classes of antipsychotics, provided that sufficient papers reporting these effects were detected.

### Assessment of certainty of evidence

We used the Grading of Recommendations Assessment, Development and Evaluation (GRADE) framework to assess the certainty of the evidence from the reviews and the strength of the recommendations [23] This approach identifies four elements which influence the certainty of the evidence: study design, study quality (risk of bias), consistency (between estimates of effect across reviews) and directness (i.e. applicability of participants, interventions, comparisons and outcomes of included reviews to the clinical question under review).

## Results

**Fig. 1:**
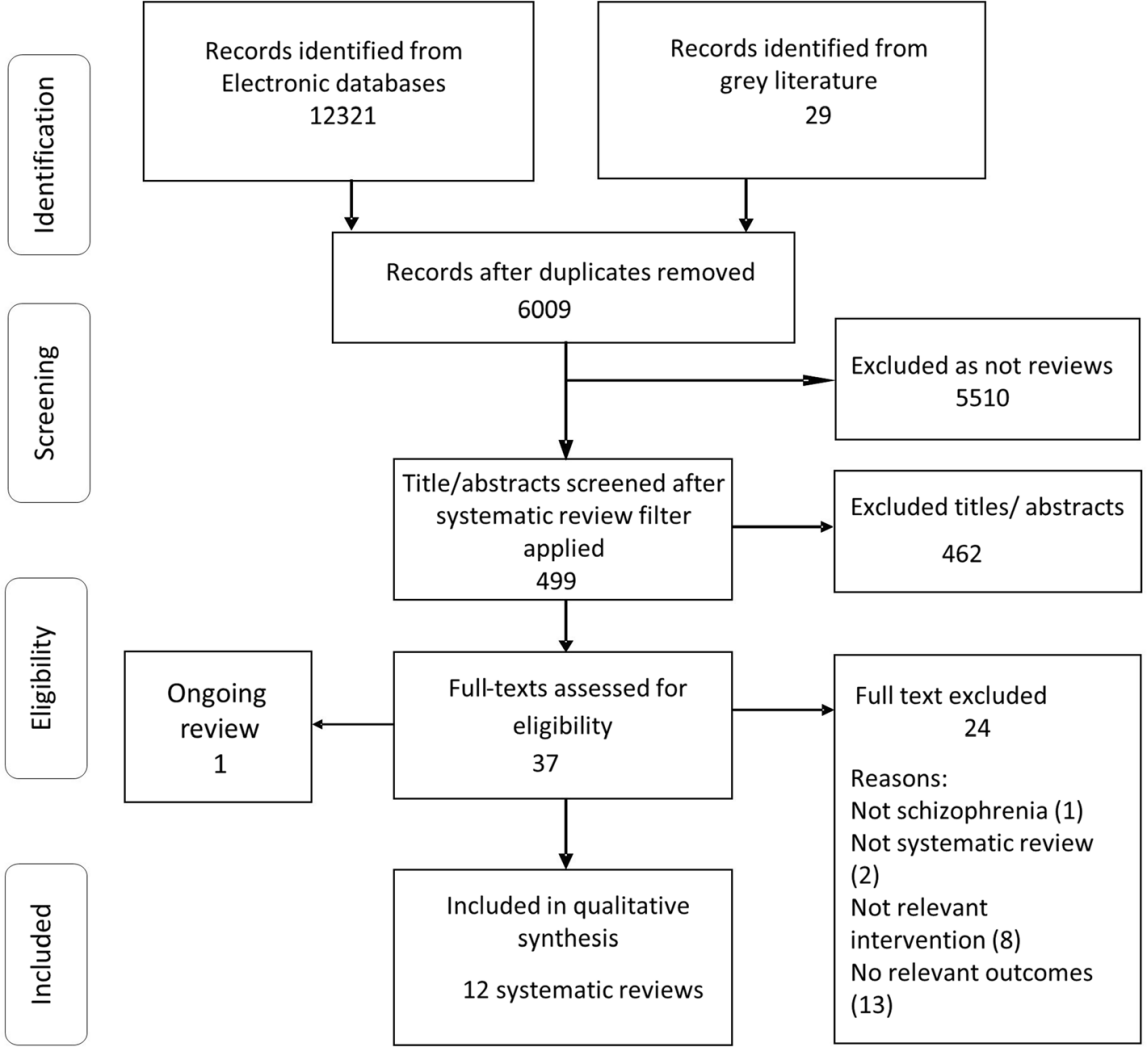
PRISMA flow chart of review selection process

The multiple database search located 12,321 citations. Complementary searching (see web appendix for details) resulted in 29 further unique citations. Removing 6341 duplicates and applying the systematic review filter resulted in 499 references. Thirty-seven of these were assessed in full text and 12 were included. One ongoing review was also identified. (See the PRISMA flow chart in Fig. 1). This review and the excluded full text reviews with reasons are reported in web appendix (Additional file 2). The most common reason for exclusion was lack of any metabolic syndrome outcomes (*n* = 13) followed by the review not addressing APP (*n* = 8), and opinion articles (*n* = 2). One review did not address schizophrenia.

We also extracted data on BMI from the included reviews. These additional data are available in a web appendix (Additional file 3).

### Description of included reviews

Twelve systematic reviews were included [24–35]. The numbers of primary studies included in the reviews ranged from 5 to 72 (median 46), although this number was not always reported. When this was the case we derived the figure from the tables and forest plots in the paper. One ongoing Cochrane review was also identified [36]. All except two reviews reported search dates. Most were from the date of inception of the databases or, in one case from 1985. The most recent searches were up until 2015 in two reviews.

The inclusion criteria in the reviews varied and methods used for inclusion were often not explicitly described. (Table 1). All reviews included diagnosed schizophrenia patient populations on antipsychotic therapy. APP was compared to antipsychotic monotherapy in six reviews. The other six did not specify the comparison. Outcomes of interest that were reported in the included reviews were lipid metabolism markers (8 reviews), diabetes or glucose metabolism markers (3 reviews), and hypertension (1 review).

**Table 1:** Characteristics of included systematic reviews on effects of APP

Study	Studies (N) included	Date of latest search	Study Settings (Primary/ Secondary care)	Included Study Designs	Participants	Intervention/Exposures	Comparison	Outcomes	Quality assessment in the review	Meta-analysis	Findings/ effect ^a^on metabolic markers/ conditions
Galling 2016 [32]	67	05/25/2015	Both	RCTs	Schizophrenia /schizoaffective disorder	Any APP; APP with D2 antagonists; APP with partial D2 agonists	AP monotherapy	Total cholesterol; LDL cholesterol;	No formal assessment; all studies considered at high risk; sensitivity analysis done for blinding.	Yes	APP was associated with lower total cholesterol and LDL-cholesterol
Young 2015 [25]	53	June 2013	NR	Case control; cohort; cross-sectional	All diagnostic groups. Children and adults	Any AP	AP monotherapy	Prevalence of: Metabolic disorder; diabetes mellitus; hypertension;	Author defined criteria used to give scores and only highest scoring studies summarised.	No	APP was associated with increased prevalence of AE: metabolic syndrome, dyslipidaemia, diabetes. A longer duration of treatment was associated with greater severity; Clozapine strongly associated with metabolic disturbance
Tranulis 2008 [26]	51	23 Aug 2006	NR	All	Schizophrenia	APP specific combinations	NR	Safety	NR	No	No synthesis^b^/effect estimate on metabolic outcomes.
Tracy 2013 [27]	72	6 January 2013	NR	All	Schizophrenia/schizoaffective disorder or related diagnoses	APP	NR	Any functional outcome or adverse effect	NR	No	No synthesis/effect estimate on metabolic outcomes. They report that there is consistent emerging data supporting aripiprazole for reregulate lipid profiles.
Canadian Agency for Drugs and Technologies in Health 2012 [35]	30	June 16, 2010	NR	RCT	schizophrenia /schizoaffective disorder inadequately managed with one or more AAPs at recommended doses	High dose AP/ APP	AP Low dose /monotherapy	AE (endocrine / metabolic markers for glucose, prolactin,	Modified SIGN checklist assigned a rating (very good, good, or poor); 27/30 rated poor. Low quality tested in sensitivity analyses	Probably yes but results NR	Total cholesterol and LDL were statistically significantly lower with Clozapine +AP.
Zheng 2016 [24]	55	June 2015	NR	RCT	Patients using antipsychotic drugs	Aripiprazole + AP	Placebo + AP or AP alone	No outcome of interest	Cochrane Risk of bias tool: 2 trials at low risk	Yes	No effect estimate on metabolic outcomes.
Mizuno 2014 [29]	50	November 5, 2013	NR	RCT	schizophrenia or related psychotic disorders	NR; likely any APP	AP plus placebo or AP monotherapy	HbA1C, LDL, total cholesterol	Cochrane Risk of bias tool: results NR	Yes	Add-on aripiprazole compared to monotherapy led to l better HbA1C control, and better lipid profile.
Lerner 2004 [31]	29	2003	Both	All	treatment-resistant t schizophrenic /schizoaffective patients on combinations atypical antipsychotics	Typical AP+ Atypical AP	AP	AE	NR	No	No synthesis; No effect reported for metabolic outcomes.
Srisurapanont 2015 [28]	5	July, 2014	NR	RCT	Schizophrenia patients with unsatisfactory response to clozapine	Aripiprazole + clozapine	Clozapine alone (AP)	AE (LDL)	Cochrane Risk of bias tool: 1 trial at low risk in all domains	Yes	LDL reduction effects favoured APP with aripiprazole addition compared to monotherapy.
Gallego 2012 [33]	16	NR	NR	All	NR	APP in general; specific APP combinations	NR	AE (glucose and lipid metabolism effect/ metabolic syndrome)(not defined)	NR	No	No synthesis; expert opinion presented: APP carries an increased side effect burden compared to monotherapy; Aripiprazole augmentation was associated with a decrease in dyslipidaemia.
Lochmann van Bennekom 2013 [30]	46	April 2012	NR	RCT; SR	Schizophrenia	APP	NR	AE	NR	No	No synthesis; no effect on metabolic outcomes.
Correll 2013 [34]	8	NR	NR	RCT	schizophrenia	APP	Placebo	glucose and lipid metabolism; cardio metabolic outcomes (not defined)	NR	Yes	Aripiprazole+ clozapine/ olanzapine led to: Significant reduction in total and LDL cholesterol and triglycerides, but not in HDL-cholesterol or glucose. No significant cardio metabolic effects were found with risperidone/ fluphenazine + clozapine, aripiprazole + quetiapine/ risperidone, or aripiprazole + haloperidol.

^a^As reported by reviews; ^b^No synthesis means there was no attempt to combine outcome data across studies and each study was individually described

*AE* Adverse Effect, *Ap* Antipsychotic Pharmacotherapy; *APp* Antipsychotic Poly Pharmacotherapy, *BMI* Body Mass Index, *BPRS* Brief Psychiatric Rating Scale, *CI* Confidence Interval, *HbA1C* Glycated Haemoglobin (A1c), *HDL* High Density Lipoproteins, *LDL* Low Density Lipoproteins, *MA* Meta-Analysis, *MD* Mean difference between groups, *N* Number of studies, *n* number of participants, *NA* Not Applicable, *NR* Not Reported, *PANSS* Positive and Negative Syndrome Scale, *RCT* Randomised Controlled Trial, *RR* Relative Risk, *SIGN* Scottish Intercollegiate Guidelines Network

Only two included reviews provided definitions for the metabolic outcomes: Mizuno et al. 2014 [29] defined metabolic outcomes (fasting glucose, HbA1C, Total LDL (Low density lipoprotein) and HDL (High density lipoprotein) cholesterol) as ‘at endpoint as defined in individual studies’. Young et al. 2015 [25] defined metabolic syndrome as either the International Diabetes Federation (IDF) criteria for metabolic syndrome in adults and children, or National Cholesterol Education Programme (NCEP) criteria for metabolic syndrome. Similarly, dyslipidaemia was defined as at least one of the followings: total cholesterol > 200 mg/dL; HDL cholesterol 120 mg/dL; triglycerides ≥150 mg/dL; treatment for a known lipid disorder.

No reviews reported useable data for subgroup analyses.

### Quality of included reviews

All the included reviews were considered at high risk of bias based on AMSTAR assessments (21). For one review (33) we could not locate a full text or a protocol and so assessments were based on abstract information alone.

In total, four reviews reported an a priori design, only two performed study selection and data extraction in duplicate, six performed a comprehensive literature search, and only five included both published and unpublished studies irrespective of language of publication.

None of the reviews provided a list of both included and excluded studies and only six provided characteristics of included studies as required by the AMSTAR criteria. Four assessed and documented the scientific quality of included studies and three of these used the scientific quality of the included studies appropriately in formulating conclusions.

Five reviews used appropriate methods to combine studies in a meta-analysis, but none provided a conflict of interest statement or funding sources of included studies, although most did report this for the authors. Most of the review authors were supported by one or more pharmaceutical companies.

Although none of the reviews were judged to be at low risk of bias, in our opinion reviews by Mizuno et al. and Anonymous et al. were more reliable because these used an a priori protocol, duplicate selection and extraction, and comprehensive searching without limits and also considered the study quality in their findings and conclusions. (See Table 2).

**Table 2:** Quality of included systematic reviews (AMSTAR)

Review ID AMSTAR Questions	Galling et al. 2016 [32]	Young et al. 2015 [25]	Tranulis et al. 2008 [26]	Tracy et al. 2013 [27]	Anonymous 2012	Zheng et al. 2016 [24]	Mizuno et al. 2014 [29]	Lerner et al. 2004 [31]	Srisurapanont et al. 2015 [28]	Gallego et al. 2012 [33]	Lochmann van Bennekom et al. 2013 [30]	Correll et al. 2013 [34]
Was an ‘a priori’ design provided?	N	N	N	Y	Y	Y	Y	N	U	N	N	U
Was there duplicate study selection and data extraction?	N	N	N	N	Y	N	Y	N	N	N	N	U
Was a comprehensive literature search performed?	N	N	N	Y	Y	Y	Y	N	Y	N	N	U
Was the status of publication (i.e. grey literature) used as an inclusion criterion?	N	N	N	Y	Y	N	Y	N	Y	N	N	U
Was a list of studies (included and excluded) provided?	N	N	N	N	N	N	N	N	N	N	N	U
Were the characteristics of the included studies provided?	Y	N	N	Y	N	Y	Y	Y	Y	N	N	U
Was the scientific quality of the included studies assessed and documented?	N	Y	N	N	Y	Y	U	N	Y	N	N	U
Was the scientific quality of the included studies used appropriately in formulating conclusions?	N	N	N	N	Y	Y	Y	N	Y	N	N	U
Were the methods used to combine the findings of studies appropriate?	Y	N	N/A	N/A	Y	Y	Y	N/A	Y	N/A	N/A	U
Was the likelihood of publication bias assessed?	Y	N	N	N	N	Y	Y	N	Y	N	N	U
Was the conflict of interest included?	N	N	N	N	N	N	N	N	N	N	N	U

*N/A* Not applicable, *N* No, *U* unclear, *Y* Yes. Note: for Correll et al. 2013 no full text was found so assessments based on abstract only

### Metabolic syndrome

Metabolic syndrome was an outcome in two reviews (Table 3). Young et al. [25] reported an association between APP and metabolic syndrome but did not provide either an estimate or a reference for the source of the data. Gallego and colleagues [33] found three studies showing increased risk of metabolic syndrome in APP (without specifying individual agents) but this association did not persist after controlling for sociodemographic and lifestyle factors.

**Table 3:** Metabolic effects of APP for schizophrenia reported in included reviews

Review ID	Outcome measure	Findings reported	Interpretation
Metabolic syndrome			
Gallego 2012 [33]	Metabolic syndrome	No synthesis or conclusion reported for this outcome	Not applicable (comparison not specified)
Young 2015 [25]	Proportion with Metabolic syndrome in APP users	No synthesis or data reported.	They report that there is an association between metabolic syndrome and APP but no data reported.
Lipid profile outcomes			
Galling 2016 [32]	Mean Total cholesterol mg/dl	SMD −0.27 (95%CI -0.43, −0.10)	APP was associated with lower total and LDL cholesterol compared to monotherapy
	Mean LDL mg/dl	SMD −0.28 (95%CI -0.45, − 0.11)	
Canadian Agency for Drugs and Technologies in Health 2012 [35]	Mean Total cholesterol	Total cholesterol statistically significantly lower with Clozapine +AP	Adding a second antipsychotic to clozapine was associated with lower total and LDL cholesterol compared to monotherapy with clozapine.
	Mean LDL	LDL statistically significantly lower with Clozapine +AP	
Tracy, 2013 [27]	NR	Aripiprazole co-treatment reregulates lipid profiles	APP including aripiprazole is associated with good lipid profile (comparison not specified)
Srisurapanont, 2015 [28]	Mean LDL mg/dl	MD −11.06 (95%CI -18.25, −3.87)	Aripiprazole + clozapine APP was associated with lower total and LDL cholesterol compared to monotherapy with clozapine
Mizuno, 2014 [29]	Mean Total cholesterol mg/dl	MD −12.81 (95%CI -19.35, −6.27)	
	Mean LDL mg/dl	MD − 11.69 (95% CI -19.12, −4.26	
Gallego, 2012 [33]	NR	Aripiprazole augmentation was associated with a decrease in dyslipidaemia	APP with aripiprazole is associated with good lipid profile (comparison not specified)
Correll, 2013 [34]	Mean Total cholesterol	SMD −0.4 (95% CI -0.7,-0.2)	APP with aripiprazole was associated with lower triglycerides, and total and LDL cholesterol but not HDL cholesterol, compared to clozapine or olanzapine monotherapy
	Mean LDL	SMD −0.3 (CI -0.6,- 0.1)	
	Mean triglycerides	SMD −0.4 (CI -0.7,- 0.0)	
	HDL level	Mean NR; *p* = 0.95	
Glucose profile outcomes			
Mizuno, 2014 [29]	Mean HbA1C	MD −0.65 (95%CI -1.25, − 0.06)	APP with aripiprazole is associated with lower HbA1C levels than AP monotherapy
Correll, 2013 [34]	Decrease in glucose levels	Mean NR; *p* = 0.41	APP with aripiprazole was associated with no significant change in glucose levels compared to clozapine or olanzapine monotherapy
Gallego, 2012 [33]	NR	No synthesis or data reported.	APP has been associated with Increased diabetes.
Hypertension			
Galling 2016 [32]	Hypertension (not defined)	SMD/RR (not defined): 0.97, 95%CI 0.32 to 2.98, *p* = 0.97	No conclusions drawn. Data indicate no difference between AP monotherapy and APP with D2 antagonists

*dl* decilitre, *HbA1C* glycated haemoglobin, *HDL* High Density Lipoprotein, *LDL* Low density Lipoproteins, *MD* mean difference, *mg* milligram, *NR* not reported, *NNT* numbers needed to treat; *p* probability value, *RR* Risk Ratio, *SMD* standardised mean difference

### Hyperlipidaemias

Seven reviews reported measures of lipid metabolism [25, 27–29, 32–35]. All reported that lipid profiles were better with APP particularly when aripiprazole was used as the augmentation drug (*n* = 6).

### Diabetes/ glucose metabolism disorder

Three reviews addressed these outcomes. Gallego et al. [33] did not report any data or conclusions on glucose levels or diabetes but reported that APP was associated with diabetes. The other two reviews reported measures of glucose metabolism [29, 34] where one [29] found a small non-significant improvement in HbA1C levels in APP involving aripiprazole compared to monotherapy and the other [34] a non-significant decrease in glucose levels with APP involving aripiprazole.

### Hypertension

Only one review provided information on hypertension [32] and reported that the effect of APP on hypertension was the same as monotherapy.

### Certainty of evidence

Applying GRADE criteria to our included reviews we found that the evidence for all the outcomes was very low quality meaning that the evidence is very uncertain. (See Table 4).

**Table 4:** GRADE table for APP compared to Antipsychotic monotherapy for metabolic effects in schizophrenia

GRADE assessment							
Outcome	№ of reviews	Risk of bias	Inconsistency	Indirectness	Imprecision	Other considerations	GRADE Quality rating
Metabolic syndrome	2	Serious^a^	Serious^b^	Serious^b^	Serious^b^	Publication bias strongly suspected^c^	⊕◯◯◯ VERY LOW
Lipid disorder	8	Serious^a^	Not serious	Serious^d^	Serious^e^	Publication bias strongly suspected^f^	⊕◯◯◯ VERY LOW
Diabetes	3	Serious^a^	Serious^g^	Serious^d^	Serious^e^	Publication bias strongly suspected strong association^h^	⊕◯◯◯ VERY LOW
Hypertension	1	Serious^a^	Not serious	Not serious	Serious^e^	Publication bias strongly suspected^i^	⊕◯◯◯ VERY LOW

Explanations

^a^All included reviews were low quality based on AMSTAR. Not all reviews included RCTs alone, and not all performed quality assessment of the included studies. Primary studies were short term, small and often uncontrolled

^b^Only one review reported findings but without data on direct comparison

^c^None of the two reviews searched for unpublished studies or assessed publication bias

^d^Reviews used various comparisons (before and after; one time prevalence; specific combinations of antipsychotic versus any antipsychotic)

^e^Wide confidence intervals and/or ranges

^f^Only two reviews searched for unpublished studies and none assessed publication bias

^g^Review findings were contrasting

^h^Only one review searched for unpublished studies. None assessed publication bias

^i^Only one review reported on this outcome which did not report a search for unpublished studies nor assessed publication bias for this outcome

Grade evidence levels

High = Further research is very unlikely to change our confidence in the estimate of effect

Moderate = Further research is likely to have an important impact on our confidence in the estimate of effect and may change the estimate

Low = Further research is very likely to have an important impact on our confidence in the estimate of effect and is likely to change the estimate

Very low = Any estimate of effect is very uncertain

## Discussion

Twelve studies fulfilled the criteria for this review of reviews. This is a large body of evidence indicating the degree of continued interest in the topic of antipsychotic polypharmacy in people with schizophrenia. In the context where there are strong opposing opinions about whether APP is harmful or beneficial, this extensive body of work shows researchers’ commitment to confirm through science what may be seen as an intuitive therapeutic approach.

Overall, our findings are in line with several of the included reviews – namely that there is insufficient evidence to clearly answer the questions on the efficacy and potential harms of APP.

In general, the quality of evidence was found to be low. This was in part likely to be due to limitations in primary studies included in the reviews. Most reviews did not include a synthesis of findings (either as a meta-analysis or a narrative synthesis) and only provided descriptions of included studies. However, where the study findings were pooled in reviews, the ranges and confidence intervals around the effect were wide, indicating uncertainty. Most reviews also did not include studies from the grey literature and did not assess publication bias. While five reviews compared APP to antipsychotic monotherapy explicitly, some of the reviews did not report their comparisons. Evidence on hypertension was limited (one review) and for all other outcomes the findings were heterogeneous across reviews.

With the exception of combinations involving aripiprazole, it was not possible to ascertain whether some combinations of antipsychotics were less harmful than others, or if associations of first generation with second generation antipsychotics had a differential effect on the selected outcomes. Six reviews [27–29, 32–34] suggested a protective effect of antipsychotic combinations which included aripiprazole for dyslipidaemia and glucose metabolism, compared to other combinations and/or monotherapy. Given the quality of evidence it would be premature to conclude that in the presence of another antipsychotic, aripiprazole protects against the risk of metabolic syndrome. Yet, the findings raise an intriguing hypothesis that warrants further investigation into why risks associated with combinations containing aripiprazole differ from those containing other commonly used atypical antipsychotics. The relationship between an antipsychotic drug’s mechanism or receptor binding profile and metabolic syndrome is thought to be very complex, and likely to be multifactorial, perhaps involving interplay of dopamine, histamine, orexigenic neuropeptides, adrenergic and muscarinic receptors, and failed glucose homeostasis, with other risk factors [37].

While aripiprazole differs from other established atypical antipsychotics by being a partial agonist at the dopamine D2 receptor, the possibility of reduced risk of metabolic syndrome in combination treatment may relate to its action on the serotonin system rather than the dopamine system. One area which has received much attention for atypical antipsychotics known to carry a relatively high risk of metabolic complications, such as clozapine and olanzapine, is their high affinity for serotonin 5-HT2C receptors. It has been postulated [38] that while aripiprazole acts on orexin and histamine systems that might be protective, its key pharmacological property may be its partial agonist activity at the serotonin 5-HT1A receptor which may counterbalance the problematic effects exerted through the 5-HT2C receptor. This benefit would apply not only in offsetting its own actions on 5-HT2C, but also those of co-prescribed antipsychotics with high affinity for this receptor.

No synthesis of effects considering dose equivalents of antipsychotics was found. The use of dose equivalents allows for comparison of dosage between different drugs. It is possible that APP is only harmful relative to monotherapy when the final equivalent dose is excessive and not when it is kept within established therapeutic ranges [13]. Some reviews [28, 31–33, 35] addressed APP which included clozapine. Clozapine can cause leukopenia [39] and should only be used after an ineffective trial with two other antipsychotics [8]. Furthermore, people treated with clozapine combinations have more chronic illness than other patients because they are considered to be treatment-resistant, and this factor may confound the occurrence of poorer physical health outcomes within this sub-group.

### Limitations

We searched multiple databases and employed complementary approaches to ensure no relevant published reviews were missed. The relatively large number of reviews detected indicates that this is an active research area. Our inclusion and search criteria were broad and therefore include heterogeneous patient populations, APP interventions (combinations), comparators and study designs. While this makes our findings generalizable, these should be interpreted with caution due to the limited data and quality of the included reviews. For example, the lack of uniform comparisons with monotherapy (specific AP drug, any AP drug, atypical or typical AP) added to problems interpreting the findings of the same outcomes across reviews. Including a broad range of systematic reviews provides a more complete picture than a single systematic review and also allows examination of any conflicting findings across this evidence base [40].

There was no evidence from the reviews on combinations involving other atypical drugs that might theoretically present a lower risk for metabolic syndrome, such as the second generation antipsychotics ziprasidone, asenapine, lurasidone and the recently introduced ‘third generation antipsychotic’ brexpiprazole. Most of these medications are relatively new in the market and more time may be needed until relevant reviews reach the publication stage. Since we did not search for primary studies we are not able to say with certainty whether this is an overlooked area in systematic reviews or in empirical research. This gap in evidence needs addressing in future to enable robust comparisons of atypical drugs available today.

Primary-care is an important part of the care pathway for schizophrenia where most stable patients are managed [41], although most prescribing of both antipsychotic monotherapy and poly-therapy is initiated in secondary care. None of the included reviews reported results separately by setting (primary and secondary). This an important gap in the evidence. Primary-care data has been used recently in long term follow up studies of psychosis treatment [42–49], however, none addressed APP and its consequences.

The evidence appeared to be of low quality for all outcomes (Table 4), partly because of the high risk of methodological and publication bias and also because the effects of APP were variable across reviews.

Although we searched for grey literature we did not request unpublished reviews or missing data from authors, which limits the comprehensiveness of our review especially if negative or inconclusive reviews are not published [50, 51]. Furthermore, we did not t search regional databases and therefore some potentially eligible reviews that are not indexed in major international databases may have been missed. However, considering the limitations in included reviews we consider that unpublished or missed reviews, if any, would also suffer from the same limitations due to the low quality primary studies. Due to the same limitations we did not pool results of the reviews statistically [52]. The lack of summarised or individual study data in full, prevented us from carrying out any additional analyses. In addition, the synthesis of review level data is complex and requires careful consideration of overlap of primary studies included in several reviews and this is not always possible or practical [40]. Therefore, we refrained from re-analysis and only relied on a narrative synthesis to derive our conclusions.

Based on the current evidence, we cannot definitively conclude that APP increases the risk for metabolic syndrome in schizophrenia, nor that it is safe, relative to antipsychotic monotherapy. It is imperative that this question is investigated in a robust prospective study before any key clinical recommendations are made. Future empirical studies should include sufficiently powered samples and adequate follow up periods with clearly defined comparison groups and outcomes to identify at risk subgroups and whether safer regimens for schizophrenia exist. Until better evidence is available, we advise that clinicians should err on the side of caution when considering prescribing APP.

## Additional files

Additional file 1: Medline search strategy. (DOC 66 kb)

Additional file 2: Tables of excluded and ongoing reviews. (DOCX 64 kb)

Additional file 3: Weight gain as reported in included reviews. (DOCX 15 kb)

## Abbreviations

AMSTAR: A MeaSurement Tool to Assess systematic Reviews
AP: Antipsychotic
APP: Antipsychotic polypharmacy
FGA: First generation antipsychotics
GRADE: Grading of Recommendations Assessment, Development and Evaluation
HbA1C: Glycated Haemoglobin (A1c)
HDL: High Density Lipoprotein
LDL: Low Density Lipoprotein
NCEP: National Cholesterol Education Programme
PROSPERO: International prospective register of systematic reviews
SGA: Second generation antipsychotics
