# Supplementary material for: Antipsychotic polypharmacy and metabolic syndrome in schizophrenia: a review of systematic reviews
Source: BMC Psychiatry. 2018 Sep 3;18:275. doi: 10.1186/s12888-018-1848-y (PMC6122457; doi:10.1186/s12888-018-1848-y)
Supplement: Supplementary file 1 — Medline search strategy. (DOC 66 kb) [file 12888_2018_1848_MOESM1_ESM.doc]

**Medline Search strategy: Antispsychotic polypharmacy in people with schizophrenia**

Database: Epub Ahead of Print, In-Process & Other Non-Indexed Citations, Ovid MEDLINE(R) Daily and Ovid MEDLINE(R) <1946 to Present>

--------------------------------------------------------------------------------

1 exp "schizophrenia and disorders with psychotic features"/ (139063)

2 (schizophreni$ or schizoaff$).ti,ab. (111482)

3 psychotic.ti. (7864)

4 (psychosis or psychoses or hebephreni$ or oligophreni$).ti,ab. (38707)

5 ((delusional or psychotic) adj2 (illness$ or disorder$)).ti,ab. (9800)

6 or/1-5 (182031)

7 letter/ (945147)

8 editorial/ (421964)

9 news/ (180823)

10 exp historical article/ (382410)

11 Anecdotes as topic/ (4740)

12 comment/ (687333)

13 case report/ (1833496)

14 (letter or comment* or case report).ti. (309801)

15 animals/ not humans/ (4301359)

16 exp Animals, Laboratory/ (793306)

17 exp Animal Experimentation/ (8249)

18 exp Models, Animal/ (484648)

19 exp rodentia/ (2944186)

20 (rat or rats or mouse or mice or animal*).ti. (1326334)

21 ((animal* adj2 (study or studies or experiment* or model*)) not human*).tw. (147919)

22 or/7-21 (8834687)

23 6 not 22 (143083)

24 ((augmentation or cotreatment or co-treatment or co-administration or combination* or combining or add-on* or supplement* or addition* or adjunct* or concurrent* or concomitant* or simultaneous* or parallel* or multiple) adj5 (antipsychotic* or anti-psychotic* or neuroleptic*)).tw. (3308)

25 ((polypharmacy or augmentation or cotreatment or co-treatment or co-administration or combination* or combining or add-on* or supplement* or addition* or adjunct* or concurrent* or concomitant* or simultaneous* or parallel*) adj3 (amisulpride or aripiprazole or asenapine or clozapine or lurasidone or olanzapine or paliperidone or quetiapine or remoxipride or risperidone or sertindole or ziprasidone or benperidol or chlorpromazine or droperidol or flupentixol or fluphenthixol or fluphenazine or haloperidol or levomepromazine or loxapine or pericyazine or perphenazine or pimozide or sulpiride or thioridazine or thiothixine or trifluperazine or zuclopenthixol)).ti. (1158)

26 ((APP or APPP or polypharmacy or polytherapy) and (antipsychotic* or anti-psychotic* or neuroleptic*)).tw. (768)

27 exp *Antipsychotic Agents/ and (Drug Therapy, Combination/ or Drug Combinations/) (4434)

28 polypharmacy/ and exp Antipsychotic Agents/ (301)

29 (("more than one" or "more than two" or ">1" or ">2") adj2 (antipsychotic* or anti-psychotic* or neuroleptic*)).tw. (630)

30 or/24-29 (8629)

31 23 and 30 (3035)

32 exp *Antipsychotic Agents/ and *polypharmacy/ (135)

33 (antipsychotic polypharmacy or anti-psychotic polypharmacy or neuroleptic polypharmacy).ti. (124)

34 32 or 33 (186)

35 34 not 22 (155)

36 31 or 35 (3073)

37 meta-analysis/ (74900)

38 meta-analysis as topic/ (15527)

39 (meta analy$ or metaanaly$ or metanaly$ or meta regression).ti,ab. (106412)

40 ((systematic$ or evidence$) adj2 (review$ or overview$)).ti,ab. (121852)

41 (reference list$ or bibliograph$ or hand search$ or manual search$ or relevant journals).ab. (34652)

42 (search strategy or search criteria or systematic search or study selection or data extraction).ab. (37351)

43 (search$ adj4 literature).ab. (41941)

44 (medline or pubmed or cochrane or embase or psychlit or psyclit or psychinfo or cinahl or science citation index or bids or cancerlit).ab. (135534)

45 cochrane.jw. (16352)

46 ((multiple treatment$ or indirect or mixed) adj2 comparison).ti,ab. (1319)

47 or/37-46 (293401)

48 36 and 47 (338)

49 36 not 48 (2735)

**Search protocol**

| **Component** | **Description** |
| --- | --- |
| Review area | **Antipsychotic polypharmacy** |
| Objectives | To identify possible risks/ adverse events associated with people with schizophrenia taking of more than one antipsychotic drug concurrently |
| Populations/aspect | People with schizophrenia |
| Interventions | polypharmacy |
| Study design | All ( SRs tagged) |
| Exclusions | Animal studies /editorials/ anecdotes/ case reports/ letters |
| How the information was searched | Databases: Medline/Premedline, Embase, Psycinfo, Cochrane Library, Web of Science  Language: all  date parameters: all |
| Search results | Medline/Premedline= 3073 (338=SR 2735=other)  Embase= 3548 (388=SR 3160=other)  Cochrane= 779 (93 SR 686 other)  Psycinfo=1809 (148 =SR 1661=other)  Web of Science=3112 (380=SR 2732=other)  Total = 12321  Total de-duplicated = 5980 ( 499=SR; 5481 =other) |
