# Supplementary material for: Antipsychotic polypharmacy and metabolic syndrome in schizophrenia: a review of systematic reviews
Source: BMC Psychiatry. 2018 Sep 3;18:275. doi: 10.1186/s12888-018-1848-y (PMC6122457; doi:10.1186/s12888-018-1848-y)
Supplement: Supplementary file 2 — Tables of excluded and ongoing reviews. (DOCX 64 kb) [file 12888_2018_1848_MOESM2_ESM.docx]

# Table of excluded reviews

| ID | Decision | Reason for exclusion |
| --- | --- | --- |
| {Westaway, 2016 #68} | Exclude | Irrelevant intervention; comparison |
| (Weinmann, Read, and Aderhold 2009) | Exclude | inappropriate outcomes – CVD, mortality not metabolic |
| (Sriretnakumar, Huang, and Muller 2015) | Exclude | CLZ and pharmacogenetics – only mention polypharmacy as a possible confounder |
| (Barnes, Whittington, and Paton 2007) | Exclude | not metabolic outcomes |
| (Ballesteros et al. 2013) | Exclude | Donepezil is not an AP |
| (Rubio et al. 2016) | Exclude | not metabolic outcomes |
| (Christy, Burnside, and Agius 2014) | Exclude | a commentary paper |
| (Jasovic-Gasic 2015) | Exclude | commentary article |
| (Zink and Dressing 2005) | Exclude | Not metabolic outcome |
| (Takeuchi et al. 2015) | Exclude | outcome is QtC interval |
| (Gierisch et al. 2014) | Exclude | not APPP but AP switching |
| (Wang et al. 2010) | Exclude | not metabolic outcomes |
| (Ogawa et al. 2014) | Exclude | acute mania |
| (Maiocchi and Bernardi 2013) | Exclude | inappropriate outcomes |
| (Hasan et al. 2012) | Exclude | not polypharmacy |
| (Chong and Remington 2000) | Exclude | not metabolic outcomes |
| (Zhornitsky et al. 2011) | Exclude | not APPP |
| (Gonzalez-Blanco et al. 2016) | Exclude | outcome prolactin and exposure not APPP |
| (Kishi, Meltzer, and Iwata 2013) | Exclude | not APPP |
| (Yu et al. 2016) | Exclude | not APPP |
| (Cotes et al. 2010) | Exclude | not metabolic outcomes |
| (Choi 2015) | Exclude | inappropriate outcomes – BWT/BMI |
| (Kontaxakis et al. 2006) | Exclude | not metabolic outcomes |
| (Galling, Roldan, and Correll 2015) | Exclude | not metabolic outcomes |

# Reference to exclude reviews

Ballesteros, J., V. Guillen, A. Zabala, B. Santos, J. R. Rueda, and I. Sola. 2013. 'Clinical outcomes in schizophrenia treated with donepezil in combination with antipsychotics', *Value in Health*, 16 (7): A542.

Barnes, T. R. E., C. Whittington, and C. Paton. 2007. 'Clozapine augmentation with another antipsychotic for treatment-resistant schizophrenia: a meta-analysis', *European Neuropsychopharmacology*, 17: S201-S01.

Choi, Y. J. 2015. 'Efficacy of adjunctive treatments added to olanzapine or clozapine for weight control in patients with schizophrenia: a systematic review and meta-analysis', *Thescientificworldjournal*, 2015: 970730.

Chong, S. A., and G. Remington. 2000. 'Clozapine augmentation: safety and efficacy', *Schizophrenia Bulletin*, 26: 421-40.

Christy, J., D. Burnside, and M. Agius. 2014. 'Combining antipsychotics; is this strategy useful?', *Psychiatria Danubina*, 26 Suppl 1: 315-21.

Cotes, R. O., D. L. Noordsy, M. McDermott, J. N. Peterson, and M. Traum. 2010. 'The risks and benefits of antipsychotic polypharmacy: Potential for Neuroleptic Malignant Syndrome', *Schizophrenia Research*, 117 (2-3): 262.

Galling, B., A. Roldan, and C. U. Correll. 2015. 'Antipsychotic polypharmacy in schizophrenia: Results from a systematic review and meta-analysis of augmentation with a second antipsychotic', *European Neuropsychopharmacology*, 25: S499.

Gierisch, J. M., J. A. Nieuwsma, D. W. Bradford, C. M. Wilder, M. C. Mann-Wrobel, A. J. McBroom, V. Hasselblad, and J. W. Williams, Jr. 2014. 'Pharmacologic and behavioral interventions to improve cardiovascular risk factors in adults with serious mental illness: a systematic review and meta-analysis', *Journal of Clinical Psychiatry*, 75: e424-40.

Gonzalez-Blanco, L., A. M. Greenhalgh, C. Garcia-Rizo, E. Fernandez-Egea, B. J. Miller, and B. Kirkpatrick. 2016. 'Prolactin concentrations in antipsychotic-naive patients with schizophrenia and related disorders: A meta-analysis', *Schizophrenia Research*, 174: 156-60.

Hasan, A., P. Falkai, T. Wobrock, J. Lieberman, B. Glenthoj, W. F. Gattaz, F. Thibaut, H. J. Moller, and Schizophrenia World Federation of Societies of Biological Psychiatry Task Force on Treatment Guidelines for. 2012. 'World Federation of Societies of Biological Psychiatry (WFSBP) Guidelines for Biological Treatment of Schizophrenia, part 1: update 2012 on the acute treatment of schizophrenia and the management of treatment resistance', *World Journal of Biological Psychiatry*, 13: 318-78.

Jasovic-Gasic, M. 2015. 'IS TREATMENT-RESISTANCE IN PSYCHIATRIC DISORDERS A TRAP FOR POLYPHARMACY?', *Psychiatria Danubina*, 27: 308-13.

Kishi, T., H. Y. Meltzer, and N. Iwata. 2013. 'Augmentation of antipsychotic drug action by azapirone 5-HT1A receptor partial agonists: a meta-analysis', *International Journal of Neuropsychopharmacology*, 16: 1259-66.

Kontaxakis, V. P., P. P. Ferentinos, B. J. Havaki-Kontaxaki, K. G. Paplos, D. A. Pappa, and G. N. Christodoulou. 2006. 'Risperidone augmentation of clozapine: a critical review', *European Archives of Psychiatry & Clinical Neuroscience*, 256: 350-5.

Maiocchi, L., and E. Bernardi. 2013. 'Optimisation of prescription in patients with long-term treatment-resistant schizophrenia', *Australasian Psychiatry*, 21: 446-8.

Ogawa, Y., A. Tajika, N. Takeshima, Y. Hayasaka, and T. A. Furukawa. 2014. 'Mood stabilizers and antipsychotics for acute mania: a systematic review and meta-analysis of combination/augmentation therapy versus monotherapy', *CNS Drugs*, 28: 989-1003.

Rubio, J. M., G. Inczedy-Farkas, S. Leucht, J. M. Kane, and C. Correll. 2016. 'Mega-review of meta-analyses investigating the short-term efficacy of pharmacologic augmentation strategies of antipsychotics in patients with schizophrenia', *European Psychiatry*, 33: S550-S51.

Sriretnakumar, V., E. Huang, and D. J. Muller. 2015. 'Pharmacogenetics of clozapine treatment response and side-effects in schizophrenia: An update', *Expert Opinion on Drug Metabolism and Toxicology*, 11: 1709-31.

Takeuchi, H., T. Suzuki, G. Remington, and H. Uchida. 2015. 'Antipsychotic Polypharmacy and Corrected QT Interval: A Systematic Review', *Canadian Journal of Psychiatry - Revue Canadienne de Psychiatrie*, 60: 215-22.

Wang, J., I. M. Omori, M. Fenton, and B. Soares. 2010. 'Sulpiride augmentation for schizophrenia', *Cochrane Database of Systematic Reviews*: CD008125.

Weinmann, S., J. Read, and V. Aderhold. 2009. 'Influence of antipsychotics on mortality in schizophrenia: systematic review', *Schizophrenia Research*, 113: 1-11.

Westaway, K., J. K. Sluggett, C. Alderman, N. Procter and E. Roughead (2016). "Prevalence of multiple antipsychotic use and associated adverse effects in Australians with mental illness." International Journal of Evidence-Based Healthcare 14(3): 104-112.

Yu, Z. H., H. Y. Jiang, L. Shao, Y. Y. Zhou, H. Y. Shi, and B. Ruan. 2016. 'Use of antipsychotics and risk of myocardial infarction: a systematic review and meta-analysis', *British Journal of Clinical Pharmacology*, 82: 624-32.

Zhornitsky, Simon, Stephane Potvin, Hoda Moteshafi, Simon Dubreucq, Pierre-Paul Rompre, and Emmanuel Stip. 2011. 'Dose-response and comparative efficacy and tolerability of quetiapine across psychiatric disorders: A systematic review of the placebo-controlled monotherapy and add-on trials', *International Clinical Psychopharmacology*, 26: 183-92.

Zink, M., and H. Dressing. 2005. '[Augmenting atypical antipsychotic medications with clozapin]', *Nervenarzt*, 76: 1092, 94-8, 100-2.

# Table of ongoing reviews

| Review ID | Last Search date | Included Study Designs | Participants | Intervention/Exposures | Comparison | Outcomes | Outcome definition | Risk of bias assessment |
| --- | --- | --- | --- | --- | --- | --- | --- | --- |
| Mayaan 2011 | June 2010 | RCT | schizophrenia or related disorders | APP | AP | AE such as weight gain: | As defined by studies | Cochrane Risk of bias tool: Results NA |

# References to ongoing reviews

MAAYAN N, SOARES-WEISER K, XIA J, ADAMS CE. Antipsychotic combinations for schizophrenia. Cochrane Database Syst Rev. 2011;2:CD009005.
