# Supplementary material for: Antipsychotic polypharmacy and metabolic syndrome in schizophrenia: a review of systematic reviews
Source: BMC Psychiatry. 2018 Sep 3;18:275. doi: 10.1186/s12888-018-1848-y (PMC6122457; doi:10.1186/s12888-018-1848-y)
Supplement: Supplementary file 3 — Weight gain as reported in included reviews. (DOCX 15 kb) [file 12888_2018_1848_MOESM3_ESM.docx]

Table of weight gain as reported in included reviews

| **Review ID** | **Outcome measure** | **Findings reported** |
| --- | --- | --- |
| Zheng, 2016 | Weight change | MD = −5.08 kg (95% CI, −7.14 to −3.02; I^2^ = 35%) |
|  | BMI | MD=−1.78 (95% CI, −2.25 to −1.31; I^2^ = 54%). |
| Young, 2015 | Proportion with weight gain% | 6–55 |
| Tracy, 2013 | Weight gain(kg) | Consistent emerging data supporting aripiprazole for helping reduce medication induced weight-gain |
| Srisurapanont, 2015 | Weight gain (kg) | MD= -1.36 (95%CI, -2.35 to -0.36) |
| Mizuno, 2014 | Weight gain(kg) | MD= −2.13 (95% CI, −2.87 to −1.39 kg; I^2^=0%) |
|  | Proportion with >7% weight loss | 13% lower in APP; NNT=9. |
| Gallego, 2012 | Weight gain (kg) | Most studies reported reduction in weight gain with APP |
| Correll, 2013 | Mean weight loss(kg) | SMD = -0.5 (95%CI, -0.8,-0.3), |
|  | Proportion with >7% weight loss | RR= 4.9 (95%CI, 1.5 to 16.4); NNT = 9 (95%CI, 6-25) |
| Anonymous, 2012 | Weight gain | No difference |
